# Impact of Resistance to Fluconazole on Virulence and Morphological Aspects of *Cryptococcus neoformans* and C*ryptococcus gattii* Isolates

**DOI:** 10.3389/fmicb.2016.00153

**Published:** 2016-02-16

**Authors:** Suélen A. Rossi, Nuria Trevijano-Contador, Liliana Scorzoni, Ana Cecilia Mesa-Arango, Haroldo C. de Oliveira, Karin Werther, Tânia de Freitas Raso, Maria J. S. Mendes-Giannini, Oscar Zaragoza, Ana M. Fusco-Almeida

**Affiliations:** ^1^Faculdade de Ciências Farmacêuticas, UNESP – Universidade Estadual Paulista, Campus Araraquara, Departamento de Análises ClíncasSão Paulo, Brazil; ^2^Centro Nacional de Microbiologia, Unidad de Micologia, Instituto de Salud Carlos III, MajadahondaMadrid, Spain; ^3^Dermatology Research Group, Universidad de AntioquiaMedellín, Colombia; ^4^Faculdade de Medicina Veterinária e Zootecnia, USP – Universidade de São Paulo, Departamento de PatologiaSão Paulo, Brazil

**Keywords:** *Cryptococcus* sp., resistance, fluconazole, virulence, *Galleria mellonella*

## Abstract

*Cryptococcus* sp. are responsible for around 1 million cases of meningitis every year. Fluconazole (FLU) is commonly used in the treatment of cryptococcosis, mainly in immunocompromised patients and the resistance is usually reported after long periods of treatment. In this study, the morphological characterization and virulence profile of FLU-susceptible and FLU-resistant clinical and environmental isolates of *C. neoformans* and *C. gattii* were performed both *in vitro* and *in vivo* using the *Galleria mellonella* model. FLU-susceptible isolates from *C. neoformans* were significantly more virulent than the FLU-resistant isolates. FLU-susceptible *C. gattii* isolates showed a different virulence profile from C. *neoformans* isolates where only the environmental isolate, CL, was more virulent compared with the resistant isolates. Cell morphology and capsule size were analyzed and the FLU-resistant isolates did not change significantly compared with the most sensitive isolates. Growth at 37°C was also evaluated and in both species, the resistant isolates showed a reduced growth at this temperature, indicating that FLU resistance can affect their growth. Based on the results obtained is possible suggest that FLU resistance can influence the morphology of the isolates and consequently changed the virulence profiles. The most evident results were observed for *C. neoformans* showing that the adaptation of isolates to antifungal selective pressure influenced the loss of virulence.

## Introduction

*Cryptococcus neoformans* and *Cryptococcus gattii* are the main etiologic agents of cryptococcosis. This infection occurs after inhalation of basidiospores, which are found in the environment (Martinez and Casadevall, 2006; Giles et al., 2009; Negroni, 2012). The clinical manifestations usually occur in pulmonary and cerebral forms and may ultimately progress to the most severe of the disease, meningoencephalitis (Chau et al., 2010; Kessler et al., 2010; Negroni, 2012). Globally, approximately one million cases of cryptococcal meningitis occur each year, the majority of which occur in sub-Saharan Africa (Park et al., 2009).

*Cryptococcus neoformans* has a worldwide distribution and mainly affects immunocompromised patients, causing high morbidity and mortality in these individuals (Li and Mody, 2010). *C. gattii* infections have historically been prevalent in regions with tropical and subtropical climates; however, outbreaks in humans and animals in temperate countries have been observed, which demonstrates that the fungus can adapt to new environments (Kidd et al., 2004; Byrnes and Heitman, 2009; Hagen et al., 2012). *C. gattii* is traditionally associated with immunocompetent individuals, but immunocompromised patients are also at risk of infection (Jobbins et al., 2010; Harris et al., 2011).

Some authors have reported that the sensitivity to azoles and virulence profiles can vary depending on the geographic region that the yeast is isolated, whether the isolate is clinical or environmental and on the molecular fungus type (Bovers et al., 2008a; Espinel-Ingroff et al., 2012a,b).

Currently, the *Cryptococcus* species complex is comprises in two species, *C. neoformans* serotypes A, D, and AD and *C. gattii* serotypes B and C (Boekhout et al., 1997, 2001; Kwon-Chung and Varma, 2006). *C. neoformans* consists of two varieties, *C. neoformans* variety *grubii* (serotype A) and *C. neoformans* variety *neoformans* (serotype D; Franzot et al., 1999). However, in the last years, several studies using molecular techniques have questioned the currently used of two species concept in the *Cryptococcus* species complex (Boekhout et al., 2001; Kidd et al., 2004; Bovers et al., 2008b; Meyer et al., 2009; Hagen et al., 2010, 2015). *C. neoformans* var. *grubii* is represented by AFLP1/VNI and two minor genotypes AFLP1A/VNII (VNB) and AFLP1B/VNII (Barreto de Oliveira et al., 2004; Litvintseva et al., 2006). *C. neoformans* var. *neoformans* is represented by AFLP2/VNIV and the hybrid (AD) by AFLP3/VNIII genotype (Trilles et al., 2003; Meyer et al., 2009). *C. gattii* is divided into five genotypes, AFLP4/VGI, AFLP6/VGII, AFLP7, and AFLP10/VGIV (serotypes B and C) and AFLP5/VGIII (Fraser et al., 2005; Hagen et al., 2010). Recently, Hagen et al. (2015) through phylogenetic and genotyping analysis, proposed to recognize the current *C. neoformans* var. *grubii* and *C. neoformans* var. *neoformans* as two separate species and, *C. gattii* in five separated species.

The increase in invasive fungal infections and the development of resistance mechanisms by some fungal species is of great concern given that antifungal treatment is usually aggressive, toxic, and inefficient (Spinello, 2013). Although the detection of resistance to antifungal agents is difficult, increased rates of fungal infections by resistant isolates and changes in breakpoints have been reported in patients exposed to long-term therapies (Fera et al., 2009; Wilke, 2011).

Virulence factors are mechanisms that allow the fungus to cause damage to the host (Casadevall and Pirofski, 1999, 2003). Many phenotypes have been specifically correlated with the virulence of *C. neoformans*, such as capsule production, melanin formation, and protein secretion. Furthermore, cell characteristics such as the cell wall and morphogenesis play an important role in host-fungus interactions (Alspaugh, 2014).

Murine animal models have been used in several studies to evaluate virulence and the efficacy of antifungal agents against different fungal species (Okawa et al., 2008); however, there is currently a particular interest in developing alternative models for studying microbial virulence (Mylonakis and Aballay, 2005; Chrisman et al., 2011; Muhammed et al., 2012). These models have provided considerable knowledge on different aspects of microbial infection (Fedhila et al., 2010) and were developed to present numerous advantages over mammalian models, such as low cost, ease of use, potential to conduct large-scale studies, and compliance with global trends regarding animal welfare and bioethics issues (Jacobsen, 2014).

*Galleria mellonella* is a lepidopteran that has been successfully used as a model to study the virulence of pathogenic fungi such as *C. neoformans* and *C. gattii* (Mylonakis et al., 2005; García-Rodas et al., 2011; Firacative et al., 2014), *Candida albicans* (Brennan et al., 2002; Rueda et al., 2014), and *Aspergillus fumigatus* (Cheema and Christians, 2011; Gomez-Lopez et al., 2014). Moreover, it has also been used to investigate the efficacy of antifungal therapy (Cowen et al., 2009; Mesa-Arango et al., 2012; Scorzoni et al., 2013).

It is known that the adaptation capacity of *C. albicans* to host environment through virulence factors can be modified in the presence or absence of a drug (Schulz et al., 2011). In bacteria, the development of antibiotic resistance mechanisms can generate a cost in adaptation (Levin et al., 1997; Andersson and Levin, 1999), especially by reducing the rate of bacterial growth (Andersson and Hughes, 2010). The impact of antifungal acquired resistance on virulence in some microorganisms, such as *A. fumigatus*, is still not well-understood. However, it is known that acquisition of resistance by specific mechanisms may result in phenotypic changes, such as, germination rate, hyphal growth, or growth rate of the resistant isolates. These changes, in turn, may result in a significant loss of virulence (Gomez-Lopez et al., 2014). These hypotheses show to us the importance further studies to better understand the relationship between the resistance to antifungal agents and the adaptation of the fungus to the host environment. Thus, in this study, we evaluated the impact of the development of resistance to fluconazole (FLU) by isolates of *C. neoformans* and *C. gattii* both on virulence and on morphological changes *in vitro* and *in vivo* using the *G. mellonella* model.

## Materials and Methods

### Strains and Growth Conditions

Three sequential clinical isolates of *C. neoformans* var. *grubii* (AFLP1VNI) were collected and classified according to the *in vitro* susceptibility to FLU as follows: resistant (30R), susceptible dose-dependent (27SDD), and susceptible (26S). Isolates were recovered from an HIV-positive patient with a history of relapses associated with treatment failure and were characterized by molecular typing and susceptibility to antifungal therapy. Additionally, the isolates were analyzed by RAPD (random amplified polymorphic DNA) and showed identical genetic profiles (data not shown). In addition, two environmental *C. gattii* isolates (isolated from a psittacine bird; AFLP6/VGII) were evaluated, one with reduced susceptibility to FLU (118R; Raso et al., 2004) and one with susceptibility to FLU (CL).The strains *C. neoformans* ATCC 90112 (AFLP1/VN1; The American Type Culture Collection) and *C. gattii* ATCC 56990 (AFLP4/VGI) were used. All species were obtained from the collection of the Laboratory of Clinical Mycology, Department of Clinical Analysis, Faculty of Pharmaceutical Sciences, UNESP, Araraquara.

### Antifungal Susceptibility

To confirm the phenotype obtained initially, antifungal susceptibility tests were conducted for all isolates using FLU and amphotericin B (Amb), according to M27-A3 document from the Clinical and Laboratory Standards Institute. The range of antifungal concentrations used was 128–0.125 μg/ml for FLU and 16–0.0321 μg/ml for Amb.

#### *Cryptococcus* sp. Growth Curve at Different Temperatures

For each assay, 10^4^ cells/mL were inoculated in 100 mL of Sabouraud broth. One milliliter of these cultures was collected after 3, 6, 9, 12, 24, 48, and 72 h and the optical density was determined at 600 nm using a spectrophotometer (Thermo Scientific Genesis). The Sadouraud broth was used as control in order to normalize readings.

#### *Cryptococcus* sp. *In Vitro* Capsule Induction

For *in vitro* morphological analysis, we induced capsule growth in all isolates as described by Zaragoza and Casadevall (2004). First, *Cryptococcus* sp. were cultivated in Sabouraud broth overnight at 30°C. Next, the inocula were concentrated in PBS (1 mL) and 100 μl were transferred to 10% Sabouraud buffered at pH 7.3 with 50 mM MOPS (3-(*N*-Morpholino) propanesulfonic acid, 4-Morpholinepropanesulfonic acid) and incubated for 24 h at 37°C with shaking at 150 rpm. Yeast cells were then suspended in India ink and photographed using a Leica DMI3000B microscope. Cell size was measured using Adobe Photoshop CS3 software. Statistics were performed by ANOVA with Tukey’s post-test.

#### *Galleria mellonella* Rearing and Larvae Manipulation

For all experiments, we used *G. mellonella* in the larval stage (Alcotán, Valencia, Spain). The larvae were fed with wax and pollen and maintained at 25°C (Ramarao et al., 2012) until reaching 0.2–0.3 g. Before infection, larvae pro-legs were cleaned with 70% ethanol and yeast suspensions were injected into larvae pro-legs using 10 μl Hamilton syringes (Hamilton, USA). Before all experiments, larvae were incubated at 30 or 37°C overnight and protected from light. Each experiment was performed in triplicate.

### Survival Assay

*Galleria mellonella* larvae were infected with 10^6^, 5 × 10^6^, 10^7^, 2.5 × 10^7^, and 5 × 10^7^ cells per larva and larval death was monitored over a period of 8 days. Lack of movement was the criterion used to define larval death. Larvae were incubated at 30 or 37°C. PBS-injected larvae and groups inoculated with *C. neoformans* ATCC 90112 of and *C. gattii* ATCC 56990 were used as controls. Statistics were analyzed using the Log-rank (Mantel–Cox) test.

### Hemolymph Melanization

The melanization assay was performed as described by Scorzoni et al. (2013). Briefly, 10 larvae were infected with 10^7^ cells/larvae and incubated at 37°C. The hemolymph was collected after 1 and 5 h of infection and 10 μl of the hemolymph was diluted 1:10 in cold PBS and centrifuged at 10,000 rpm for 5 min. The supernatants were transferred into a 96-well plate and read at 405 nm in a spectrophotometer (Thermo Scientific Genesis). PBS-injected larvae were used as a control. Statistics were performed by ANOVA with Tukey’s post-test.

#### *Cryptococcus* sp. Cell and Capsule Size Alterations After Infection in *G. mellonella*

Larvae were infected with 10^6^ cells per larva and incubated at 37°C. Immediately after infection as well as 1 and 3 days post-infection, larvae were homogenized in 100 mm pore nylon filters (Falcon, BD, USA) with 1 mL of PBS. The resulting fluid was collected and centrifuged. The cell pellet was washed (1x) and suspended in 300 μl of PBS. Subsequently, an aliquot of the cell sample was stained with India ink and visualized using a Leica DMI 3000B microscope. Adobe Photoshop CS3 software was used to measure the cells. Statistics were performed by ANOVA with Tukey’s post-test.

#### *Cryptococcus* sp. Phagocytosis Assays in *G. mellonella*

Isolates of *Cryptococcus* sp. were first stained with 10 μg/ml of calcofluor white (Sigma, St. Louis, MO, USA) for 30 min at 37°C. Then, larvae were infected with 10^6^ cells and phagocytosis was analyzed after 3 h of incubation at 37°C. Hemolymph was collected in Eppendorf tubes and diluted 1:2 in cold PBS. Phagocytosis was quantified visually using a Leica DMI 3000B microscope. Five larvae from each experimental situation were used. One hundred hemocytes from each larva were counted and the percentage of hemocytes containing *Cryptococcus* sp. internalized was calculated. Statistics were performed by ANOVA with Tukey’s post-test.

## Results

### Susceptibility Testing against Fluconazole and Amphotericin B

Antifungal susceptibility testing results showed that the *C. neoformans* isolate 30R and the *C. gattii* isolate 118R were resistant to FLU with MIC values of 64 mg/L. *C. neoformans* 27SDD (susceptible-dose dependent) showed a MIC value of 16 mg/L and ATCC 90112 and 26S were characterized as susceptible to FLU with MICs of 1 and 2 mg/L, respectively. *C. gattii* isolates CL and ATCC 56990 showed MICs of 4 mg/L. All isolates were susceptible to AmB with MIC concentrations ranging between 0.125 and 0.0625 mg/L. Throughout the work, all isolates were cultured in Sabouraud without adding FLU and even after several transfers, it became clear that resistance remained.

### Growth Curve at Different Temperatures

**FIGURE 1:**
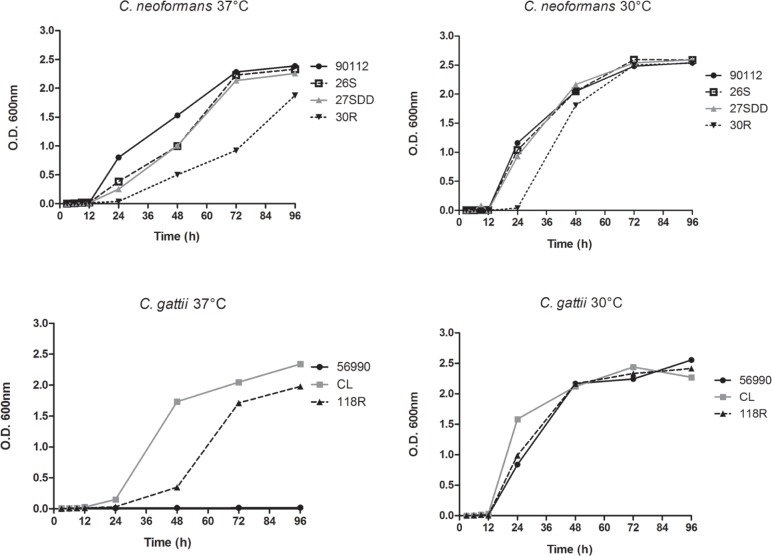
**Sequential growth curve of *Cryptococcus neoformans* isolates, *C. gattii* isolates and the ATCC strains 90112 and 56990 at 30 and 37°C**.

The assay was carried out at 30 and 37°C. *C. neoformans* and *C. gattii* showed better growth at 30°C than at 37°C. All *C. neoformans* isolates showed slower growth at 37°C, especially FLU-resistant strain 30R. When the growth curves of *C. gattii* were analyzed, it was found that isolate ATCC 56990 was unable to grow at 37°C and isolate 118R showed much lower growth compared with the susceptible isolate CL at 37°C (**Figure 1**).

#### *Cryptococcus* sp. Virulence at 30 and 37°C

**FIGURE 2:**
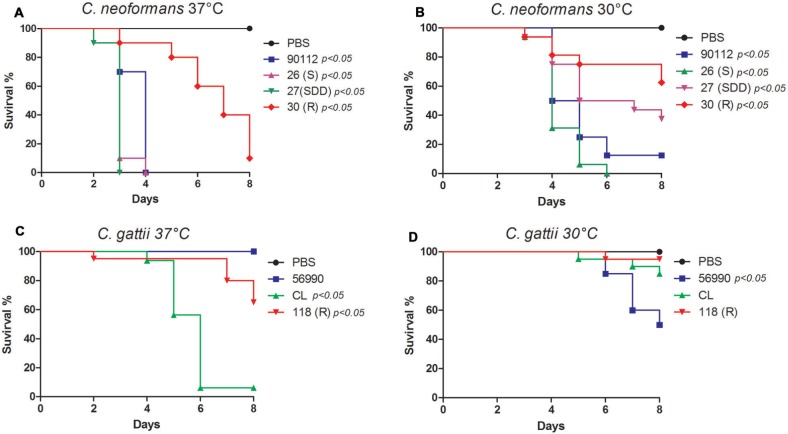
**Virulence assay in *Galleria mellonella*.** **(A)** Sequential clinical isolates of *C. neoformans*, 26S, 27SDD, and 30R incubated at 37°C; **(B)** Sequential clinical isolates of *C. neoformans*, 26S, 27SDD, and 30R incubated at 30°C; **(C)** Environmental isolates of *C. gattii* 118R and CL incubated at 37°C; **(D)** Environmental isolates of *C. gattii* 118R and CL incubated at 30°C.

Sequential clinical isolates of *C. neoformans* (26S, 27SDD, and 30R) have different virulence profiles. The thermotolerance is a very important virulence factor in *Cryptococcus* sp., therefor the virulence test was performed at 30 and 37°C. This difference was more evident between the FLU-susceptible (26S) and FLU-resistant (30R) isolates at 37°C (**Figure 2A**). At 30°C, the *C. neoformans* isolates were less virulent compared with activity at 37°C, but the virulence profiles were maintained between them with being 26S more virulent and killing 100% of the larvae at day 6 of infection (**Figure 2B**). The *C. neoformans* strain ATCC 90112 was also used in this assay and its virulence profile was very similar to the profile observed for the FLU-susceptible isolate 26S. The virulence of the *C. gattii* isolates at both temperatures was lower when compared to the *C. neoformans* isolates (**Figures 2C,D**). The strain *C. gattii* ATCC 56990 did not kill any larvae when infection was conducted at 37° (2C). The FLU-resistant isolate 118R killed 40% of the larvae, while FLU-susceptible isolate CL killed 90% of the larvae at day 8 of infection at 37°C (**Figure 2C**). At 30°C, we found a different virulence profile for *C. gattii*, when compared to the assay performed at 37°C.

To confirm the reduced virulence profile of the *C. gattii* isolates, the experiment was repeated with four additional isolates from the same species. These isolates were provided by the Mycology Reference Laboratory at the Institute of Health Carlos III of Madrid, Spain, and were identified as CL 5004 (AFLP4/VGI), CL 5010 (AFLP4/VGI), NIH191 (AFLP5/VGIII), and NIH198 (AFLP5/VGIII). Again, all *C. gattii* isolates were less virulent than *C. neoformans* (**Supplementary Figure S1**).

We also performed a survival curve with different inoculum concentrations (1 × 10^6^, 5 × 10^6^, 1 × 10^7^, 2.5 × 10^7^, and 5 × 10^7^) at 37°C. This test was performed with isolates that were less virulent with standard inoculum of the 10^6^ cells per larva in *G. mellonella* and was observed that the low virulence remained even when the inoculum was increased up to 5 × 10^7^ cells per larvae (**Supplementary Figure S2**).

### Hemolymph Melanization

**FIGURE 3:**
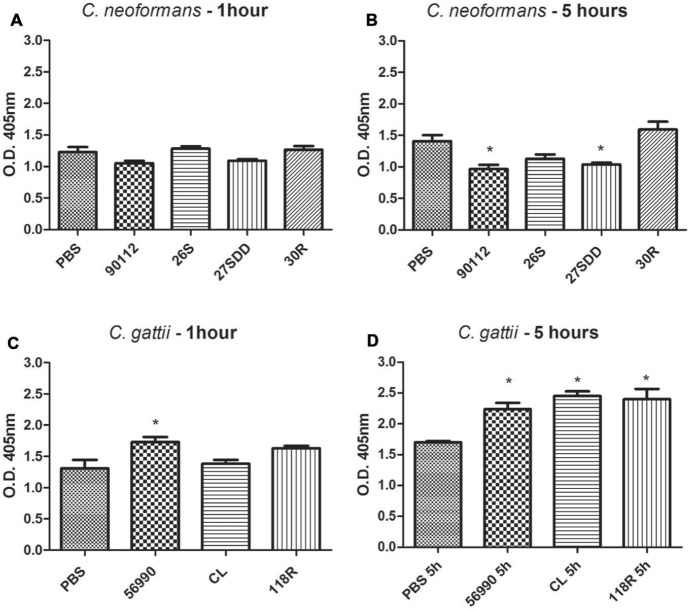
**Quantification of melanization of *C. gattii* and *C. neoformans* isolates after inoculation in *G. mellonella*.** The quantification was measured at different times, 1 and 5 h. **(A)** *C. neoformans* 1 h; **(B)** *C. neoformans* 5 h; **(C)** *C. gattii* 1 h; and **(D)** *C. gattii* 5 h. ^∗^*p* < 0.05.

During the virulence assays in *G. mellonella*, we observed significant melanization of larvae after infection with some isolates. Because of these findings, quantification of melanization in the hemolymph of the larvae was performed to verify this phenomenon. The results showed that the infection of larvae with all *C. gattii* isolates led to a significant melanin production after 5 h of infection (**Figure 3D**). However, as shown in **Figure 3B**, only the ATCC 90112 and 27SDD isolate of *C. neoformans* produced significant melanization after infection. In **Figures 3A,C** is observed that after 1 h of infection there was no significant melanization for most of the isolates, except for ATCC 56990 strain.

#### *In Vitro* Capsule Induction at 37°C

**FIGURE 4:**
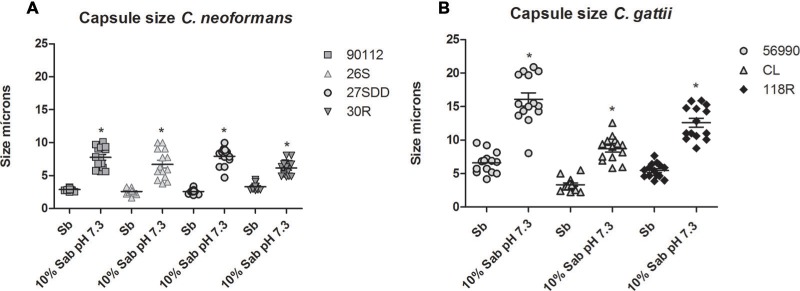
***In vitro* capsule induction of clinical isolates of *C. neoformans* and *C. gattii* environmental isolates.** **(A)** ATCC 90112 and clinical isolates of *C. neoformans* 26S, 27SDD, and 30R; **(B)** ATCC 56990 and environmental isolates of *C. gattii* CL and 118R. ^∗^*p* < 0.05.

We investigated whether resistance to FLU was associated with cell and capsule increase. After 24 h of incubation in capsule-inducing medium, all sequential clinical isolates of *C. neoformans* increased capsule size compared to the cells incubated in Sabouraud (*p* < 0.05, **Figure 4A**). When comparing the FLU-resistant isolate (30R) with the other isolates, the changes in 30R capsule size were more discrete compared to the ATCC 90112 and 27SDD isolates (*p* < 0.05).

For *C. gattii* isolates, we observed that the cells of the environmental isolates and of ATCC 56990 had greater capsule induction (*p* < 0.05, **Figure 4B**) after incubation in capsule-inducing medium. The FLU-resistant 118R isolate achieved a remarkable increase in capsule size and showed a less significant change in capsule size only when compared with the strain ATCC 56990 (*p* < 0.05).

#### Morphology of Cells in *G. mellonella*

**FIGURE 5:**
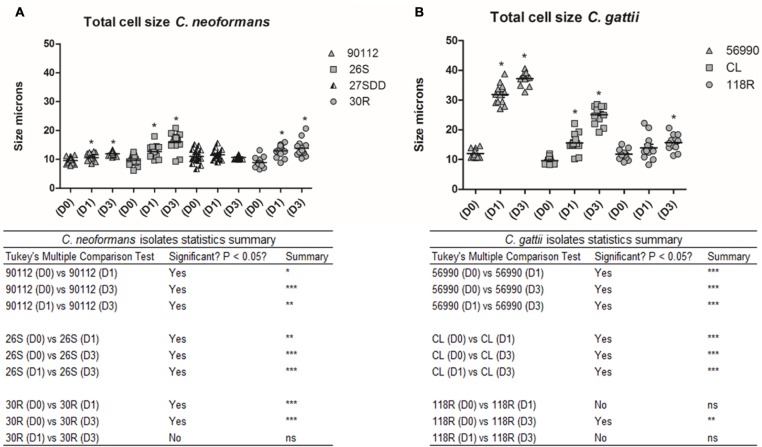
**Total cell size in microns for *C. neoformans* and *C. gattii* isolates during infection in *G. mellonella*.** The cells were recovered from *G. mellonella* at different times (D1 and D3) and controls were grown in Sabouraud (D0). **(A)** ATCC 90112 and sequential clinical isolates 26S, 27SDD, and 30R of *C. neoformans*; **(B)** ATCC 56990 and isolates CL and 118R of *C. gattii*. *^∗^p* < 0.05; *^∗∗^p* < 0.01; *^∗∗∗^p* < 0.001.

The total cell size and capsule size were measured before infection (Day 0) as well as 24 h (Day 1) and 72 h (Day 3) after infection in *G. mellonella*. A progressive increase in cell size was observed in *C. neoformans* isolates across the specified time intervals (D1 and D3) when compared with Day 0 (D0; *p* < 0.05), with the exception of the 27SDD isolate, which showed no significant changes after infection (**Figures 5A** and **7A–D**). When the isolates were analyzed individually, it was found that the FLU-susceptible isolate 26S and the ATCC 90112 strain also showed a significant increase between D1 and D3 (*p* < 0.05). For *C. gattii* isolates, a significant increase in the cell size of ATCC 56990 and CL was observed at D1 and D3 in comparison to D0 (**Figures 5B** and **7E–G**). The largest increase was observed in *C. gattii* strain ATCC 56990, where some cells reached 40 μm at D3 (**Figure 7E**). FLU-susceptible CL isolate achieved significant growth. The FLU-resistant 118R isolate only showed a significant increase at D3, however, no cells larger than 20 μ were observed (*p* < 0.05; **Figure 7G**).

**FIGURE 6:**
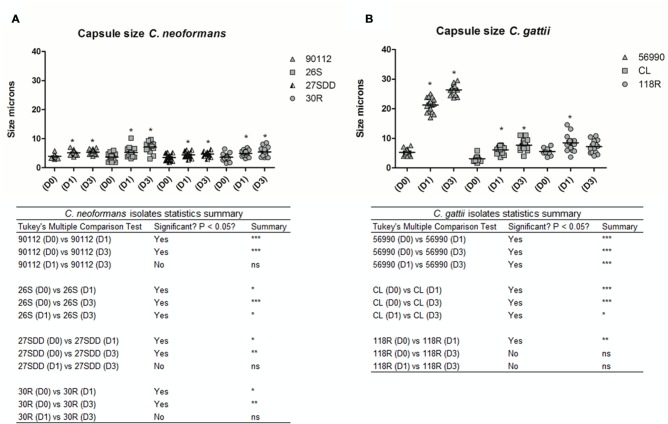
**Capsule size in microns of isolates of *C. neoformans* and *C. gattii* after infection in *G. mellonella*.** The cells were recovered from *G. mellonella* at different times (D1 and D3) and controls were grown in Sabouraud (D0). **(A)** ATCC 90112 and isolates 26S, 30R, and 27SDD of *C. neoformans*; **(B)** ATCC 56990 and isolates CL and 118R of *C. gattii*. *^∗^p* < 0.05; *^∗∗^p* < 0.01; *^∗∗∗^p* < 0.001.

**FIGURE 7:**
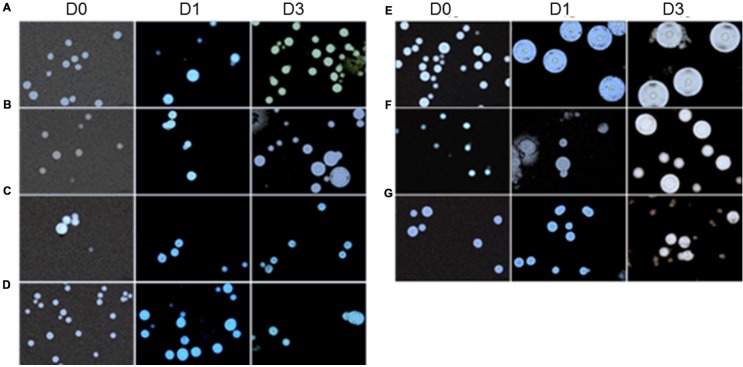
**Microscopy of *C. neoformans* and *C. gattii* isolates with India ink stain before (D0) and after infection in *G. mellonella* (D1, 24 h) and (D3, 72 h); **(A)***C. neoformans* ATCC 90112; **(B)***C. neoformans* isolate 26S; **(C)***C. neoformans* isolate 27SDD; **(D)***C. neoformans* isolate 30R; **(E)***C. gattii* ATCC 56990; **(F)***C. gattii* isolate CL; and **(G)***C. gattii* isolate 118R**.

We also measured the capsule size of the different isolates. As shown in **Figures 6A** and **7A–D**, all *C. neoformans* isolates presented a significant increase in capsule size after infection when compared with D0 (*p* < 0.05) but only 26S showed a significant and gradual increase. *C. gattii* isolates had a much greater capsule size than *C. neoformans* isolates (see **Figures 6B** and **7E–G**). The cells from *C. gattii* ATCC 56990 strain had a greater change in size after infection in *G. mellonella*. Most cells of this strain reached a capsule size ranging between 20 and 30 μ (*p* < 0.05). The capsule of environmental FLU-susceptible CL isolate presented a gradual increase at all-time intervals (*p* < 0.05) and the 118R isolate had a significant increase only at D1 (*p* < 0.05).

#### *In Vivo* Phagocytosis Assay

**FIGURE 8:**
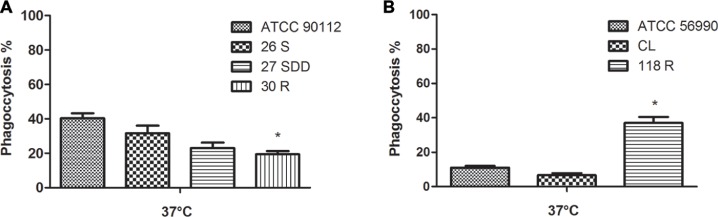
**Phagocytosis index after infection in *G. mellonella*.** **(A)** ATCC 90112 and isolates 26S, 27SDD, and 30R of *C. neoformans*; **(B)** ATCC 56990 and isolates 118R and CL of *C. gattii. ^∗^p* < 0.05.

To better elucidate the mechanisms of virulence of the selected isolates, we also performed a phagocytosis assay in *G. mellonella*. This assay showed that *C. neoformans* 30R isolate was phagocytosed to a lower degree compared with the ATCC 90112 (*p* < 0.05) strain. Among the other sequential clinical isolates, 26S, 27SDD and 30R, there was no significant difference observed in phagocytosis (**Figure 8A**). The *C. gattii* 118R isolate was the strain better phagocytosed, with almost a 40% phagocytosis rate compared to the CL and ATCC 56990 strains after infection in *G. mellonella* (see **Figure 8B**).

## Discussion

Fluconazole achieves good penetration into the central nervous system (CNS) and has minimal side effects; for this reason, FLU is commonly used in the treatment of cryptococcosis, mainly in immunocompromised patients (Brandt et al., 2001). However, in some strains, repeated exposure or prolonged treatment with azoles can lead to the development of resistance (Sanglard, 2002; Bicanic et al., 2009; Fera et al., 2009).

It is known that the development of azole resistance in fungi may occur through overexpression or mutations in the *ERG11* gene, which encodes the enzyme lanosterol 14-α demethylase (ERG11p; Sanglard et al., 1998; Cowen et al., 2000; Rodero et al., 2003; Sheng et al., 2009; Oliveira Carvalho et al., 2013), and/or by overexpression of plasma membrane proteins that pump the drug out of the cells (Posteraro et al., 2003; Sanguinetti et al., 2006; Sanglard et al., 2009). In addition, the phenomenon of heteroresistance to FLU, an adaptive mechanism of drug tolerance, has been previously described in *C. neoformans* and *C. gattii* species (Sionov et al., 2009, 2010; Varma and Kwon-Chung, 2010).

In addition to the eight major molecular types identified from molecular techniques (*C. neoformans*, VNI, VNII, VNIII, VNIV, and *C. gattii*, VGI, VGII, VGIII e VGIV), VGII has received increased interest by different authors in recent years due to the appearance of the subtypes, VGIIa, VGIIb e VGIIc (Byrnes et al., 2010; Chong et al., 2010; Firacative et al., 2014), and the discovery that susceptibility to FLU varies depending on the molecular type. Other epidemiological studies have also found that, unlike isolates from Europe and North America, yeast species originating from Africa, the Middle East, Asia-Pacific, and parts of Latin America shows variable sensitivity to FLU (Pfaller et al., 2009). The relationship between FLU resistance and virulence in *Cryptococcus* sp. has been investigated but remains poorly understood. Sionov et al. (2009) found that strains of *C. neoformans* expressing FLU heteroressistance *in vitro* (MIC ≥ 32 μg/ml) were more virulent in mice when compared with more sensitive strains (MIC ≤ 8 μg/ml). *In vivo* data have also demonstrated that a strain of *C. neoformans* with overexpression of the *AFR1* gene had significantly increased virulence concurrently with resistance (Sanguinetti et al., 2006). In the present study, clinical isolates of *C. neoformans* developed resistance to FLU possibly during patient therapy and virulence profiles were inversely proportional to the resistance, suggesting that adaptation to the selective pressure of the drug can lead to a decrease in virulence. We also found that the virulence of the *C. neoformans* 30R isolate is dependent on the concentration of inoculum, which was not observed in the isolate of *C. gattii* 118R.

Some studies show that environmental isolates of *C. gattii* have a low susceptibility to FLU (Chowdhary et al., 2011), however, the influence of FLU resistance on virulence in this species is not known. Varma and Kwon-Chung (2010) reported that *C. gattii* isolates showed heteroresistance to FLU, but the virulence was similar to those observed in isolates of *C. neoformans*. The *C. gattii* isolates in our study presented a low virulence profile in *G. mellonella*, regardless of sensitivity to FLU.

Confirming our results, recently, Santos et al. (2014), through the induction of FLU resistance in *C. gattii* isolates, observed that after the resistance induction, the virulence was diminished even *in vivo* as *in vitro* experiments. This low virulence profile was associated with the low activity of laccase and urease, a decrease in diameter and poor capsule formation (Santos et al., 2014).

Currently, it is known that morphological changes can also be considered as a virulence factor because the pathogen can evade immune recognition of the host and spread infection (Coelho et al., 2014). Nosanchuk et al. (1999) presented results showing the influence of Amb and FLU in cell morphology in *C. neoformans*. Using scanning electron microscopy, they found that cells cultured in medium with Amb or FLU in inhibitory concentrations showed a modified capsular appearance and were lower smaller when compared with cells cultured without these antifungals. Other studies have demonstrated that the occurrence of pseudo hyphae in *C. neoformans* increases its resistance to the environment but decreases its virulence *in vivo* (Magditch et al., 2012). Another type of morphological change is the appearance of *C. neoformans* giant cells, and several studies have demonstrated that this phenomenon occurs both *in vitro* and *in vivo* (Okagaki et al., 2010; Zaragoza et al., 2010; García-Rodas et al., 2011). In our results, we observed that both changes in cell size and in capsule size were detected in the majority of isolates, especially after *in vivo* infection. However, the increase in cell size of the *C. gattii* isolates (ATCC 56990 and CL) did not change the virulence, probably because the increased size of these cells was decisive for faster recognition of the immune system of *G. mellonella*, and the infection was contained. The FLU-susceptible *C. neoformans* isolate 26S was more virulent in *G. mellonella*, probably because of capsule increase after *in vivo* infection and efficient growth at 37°C. Another example of the adaptation of the fungus to high drug concentrations leading to a less virulent *in vivo* profile is paradoxical growth. This phenomenon is the ability of *C. albicans* cells to grow at high equinocandin concentrations while still fully susceptible to intermediate concentrations (Stevens et al., 2004). A study by Rueda et al. (2014) showed that this phenomenon can be an adjustment mechanism of fungus to high concentrations of caspofungin, leading to morphological changes and the rapid recognition by the immune system of the larvae. The cells maintained in high concentrations of caspofungin, influenced directly in the virulence in *G. mellonella*.

The growth at host body temperature is a requirement for virulence (Perfect, 2006), and in *C. neoformans*, the thermotolerance may have a disproportionate importance over any other virulence factor. When comparing *C. neoformans* with other species, many factors involved in virulence, such as the capsule and the production of laccase, make these species non-pathogenic because they cannot grow at the temperature of the host (Petter et al., 2001). Garcia-Solache et al. (2013) found that non-pathogenic strains of *Cryptococcus* were temperature-intolerant, and greater virulence in *G. mellonella* depended on cell concentration, whereas for *C. neoformans* var. *grubii*, the virulence increased at higher temperatures, showing that the rapid replication at 32°C can be responsible for increased virulence. The increased virulence of *C. neoformans* var. *grubii* at higher temperatures than the optimal growth temperature, could be a consequence of an increase in the stress response of the fungus, making it better able to survive in hostile conditions (Brown et al., 2007). Analyzing the growth curves of the isolates of *C. neoformans* showed that at 37°C, the FLU-resistant isolate grew less when compared to FLU-susceptible isolate, suggesting that, the resistance to FLU can somehow affect the growth at 37°C these isolates. In *C. gattii* the same was observed, but only in relation to isolate CL (sensitive FLU), because the ATCC 56990 strain was unable to grow at this temperature. The lack of growth at 37°C combined with the significant increase of cells upon infection in *G. mellonella*, may explain the virulence profile presented by the ATCC56990 strain, and low phagocytosis index. Further studies are necessary to confirm this phenotype.

Its known that genotypic differences can influence not only the virulence pattern of isolated, but in the sensitivity to azoles and clinical manifestations developed by pathogens. The phenotype observed in strain ATCC 56990 (AFLP4/VGI) can be related to genotype, however, more studies are needed to confirm this hypothesis, once differences in growth patterns, virulence and sensitivity are not linked only to genotypes and involves multiple factors. In recent study by Firacative et al. (2014) they observed that the virulence is not specifically related to a large molecular type of *C. gattii*, but with individual attributes.

Correlations between *C. gattii* molecular type and virulence pattern have been performed (Kidd et al., 2004; D’Souza et al., 2011). However, evaluations of virulence profile, in a global manner, cannot produce consistent results, suggesting that the molecular type is not crucial to the virulence of the strain (Chen et al., 2014).

The size of infectious particles can also influence virulence in *G. mellonella*. External particles are recognized by hemocytes, and at this stage, the result of the cellular immune response depends on the size of the particles. Small targets are phagocytosed while large targets are encapsulated and attacked by hemocytes (Lavine and Strand, 2002). Melanin plays an important role in the defense of *G. mellonella* and other invertebrate organisms. The humoral response in *G. mellonella* is involved in the production of various molecules with antimicrobial properties, including phenol oxidase enzyme (Eisenman et al., 2014). Melanization is a reaction catalyzed by the enzyme phenol oxidase, and it encapsulates foreign particles surrounding *G. mellonella* (Bidla et al., 2009). In our study, we observed that larvae melanization occurs when infected with *C. gattii* isolates in the first 24 h and did not occur after infection with *C. neoformans* isolates. These results are in agreement with the results found by Trevijano-Contador et al. (2015). These results suggest that recognition of isolated *C. gattii* cells by the immune system was more efficient than *C. neoformans*, probably due to cell size, increasing the survival of larvae. Scorzoni et al. (2013) found that melanization after infection with *C. krusei* was significant in *G. mellonella* and that the degree of the melanization was dependent on inoculum concentration and not on cell viability, indicating that melanization is a non-specific process that depends on the presence of foreign particles.

Our results also demonstrated that FLU-suceptible isolates of *C. neoformans* were more phagocytized after infection in *G. mellonella* and were still more virulent when these were compared with the FLU resistant isolates. These data suggest that our isolates can multiply within hemocytes, continuing infection after internalization because sensitive strains possess this ability (Santos et al., 2014). It seems that *C. neoformans* does not cause a reduction in the number of hemocytes in the first 2 h after infection (Mylonakis et al., 2005; García-Rodas et al., 2011), and this phenomenon may be related to the fact that this fungus is a facultative intracellular pathogen and can survive in phagocytic cells without affecting their viability (Feldmesser et al., 2001; García-Rodas and Zaragoza, 2012). *C. neoformans* behaves as a facultative intracellular pathogen in mammals and in other non-mammalian hosts (Diamond and Bennett, 1973; Steenbergen et al., 2001; Alvarez and Casadevall, 2006; Ma et al., 2006; Qin et al., 2011), and recently, Trevijano-Contador et al. (2015) demonstrated that the fungus is also able to multiply within hemocytes, suggesting that this ability may be another virulence factor. The FLU sensitive isolates of *C. gattii* were less phagocytosed when compared to the same drug resistant isolate, probably due to the increase of the capsule and the cell body as observed after infection in *G. mellonella*.

Fluconazole and other azole antifungal agents are widely used to prevent and treat infections caused by *C. neoformans* and *C. gattii*. Their proven efficacy and safety combined with their excellent pharmacokinetic profiles make these agents extremely important in the management of cryptococcal infections (Sanguinetti et al., 2006), but the development of resistance mechanisms and the low susceptibility of some isolates may make treatment difficult to maintain, mainly in countries where the mortality rates for these etiologic agents are high.

There are clinical differences between infections of *C. neoformans* and *C. gattii*, and treatment for longer periods in patients affected by *C. gattii* species led better results (Marr, 2011; Ngamskulrungroj et al., 2012; Chen et al., 2013). Therefore, understanding the mechanisms responsible for these phenotypic and clinical differences is extremely important because the expression of different virulence factors associated with each species is still poorly understood. Virulence studies conducted mainly with *C. neoformans* are essential, but it is difficult to extrapolate results from these tightly controlled studies to clinical isolates where multiple virulence determinants are expressed in various quantities in a coordinated and potentially host-dependent manner (Clancy et al., 2006). Cryptococcosis remains a difficult management issue, with little new drug development or recent definitive studies (Perfect et al., 2010).

How FLU resistance influences virulence is a complex issue that needs further study. However, the data obtained in this study clearly demonstrate that the adaptation of the fungus to the stress produced by the drug leads to loss of virulence, and morphological changes are involved in the production of this phenotype.

## Author Contributions

SR, OZ, and AF-A conceived and designed the experiments. SR, NT-C, LS, AM-A, and HdO performed the experiments. SR, LS, HdO, OZ, MM-G, and AF-A analyzed the data. SR, NT-C, LS, AM-A, HdO, KW, TdFR, MM-G, OZ, and AF-A drafted the manuscript. All authors read and approved the final manuscript.

## Conflict of Interest Statement

The authors declare that the research was conducted in the absence of any commercial or financial relationships that could be construed as a potential conflict of interest.
