# Corrigendum: Impact of Resistance to Fluconazole on Virulence and Morphological Aspects of *Cryptococcus neoformans* and *Cryptococcus gattii* Isolates

**DOI:** 10.3389/fmicb.2016.00537

**Published:** 2016-04-22

**Authors:** Suélen A. Rossi, Nuria Trevijano-Contador, Liliana Scorzoni, Ana C. Mesa-Arango, Haroldo C. de Oliveira, Karin Werther, Tânia de Freitas Raso, Maria J. S. Mendes-Giannini, Oscar Zaragoza, Ana M. Fusco-Almeida

**Affiliations:** ^1^Laboratório de Micologia Clínica e Núcleo de Proteômica, Faculdade de Ciências Farmacêuticas, Universidade Estadual Paulista Júlio Mesquita FilhoAraraquara, Brazil; ^2^Unidad de Micología, Centro Nacional de Microbiología, Instituto de Salud Carlos IIIMajadahonda, Spain; ^3^Department of Dermatology Research, Universidad de AntioquiaMedellín, Colombia; ^4^Departamento de Patologia Veterinária, Faculdade de Ciências Agrárias e Veterinárias FCAV/UNESP, Universidade Estadual PaulistaSão Paulo, Brazil; ^5^Departamento de Patologia, Faculdade de Medicina Veterinária e Zootecnia, Universidade de São PauloSão Paulo, Brazil

**Keywords:** *Cryptococcus* spp., resistance, fluconazole, virulence, *Galleria mellonella*

Due to an oversight on our part the affiliation of author Karin Werther is incorrect. The correct affiliation is: Faculdade de Ciências Agrárias e Veterinárias FCAV/UNESP, Departamento de Patologia Veterinária, Universidade Estadual Paulista, Jaboticabal, Brazil.

The authors apologize for this mistake. The correction does not affect the scientific validity of the results.

## Author contributions

All authors listed, have made substantial, direct, and intellectual contribution to the work, and approved it for publication.

### Conflict of interest statement

The authors declare that the research was conducted in the absence of any commercial or financial relationships that could be construed as a potential conflict of interest.

