# Supplementary figures and images for: Impact of Resistance to Fluconazole on Virulence and Morphological Aspects of Cryptococcus neoformans and Cryptococcus gattii Isolates
Source: Front Microbiol. 2016 Feb 16;7:153. doi: 10.3389/fmicb.2016.00153 (PMC4754443; doi:10.3389/fmicb.2016.00153)

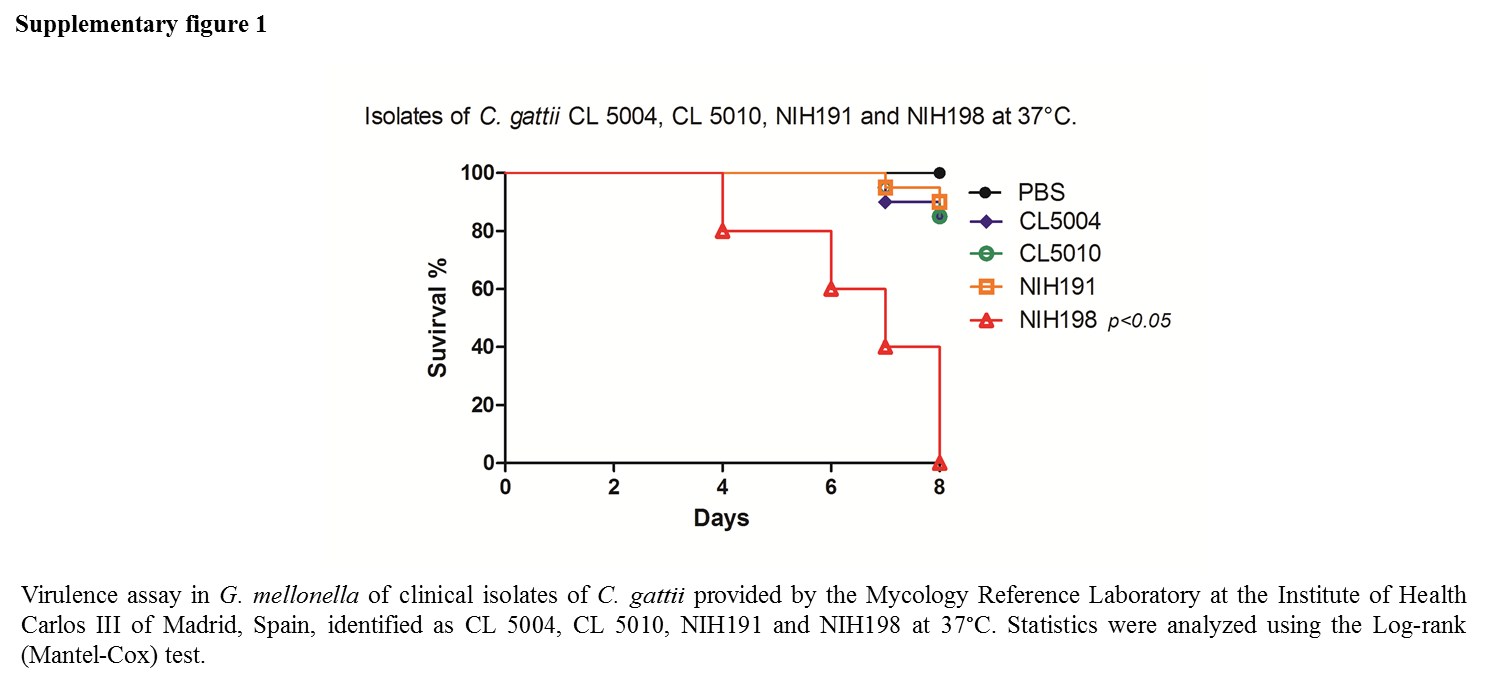

Supplement: Supplementary file 1 [file Image_1.TIF]

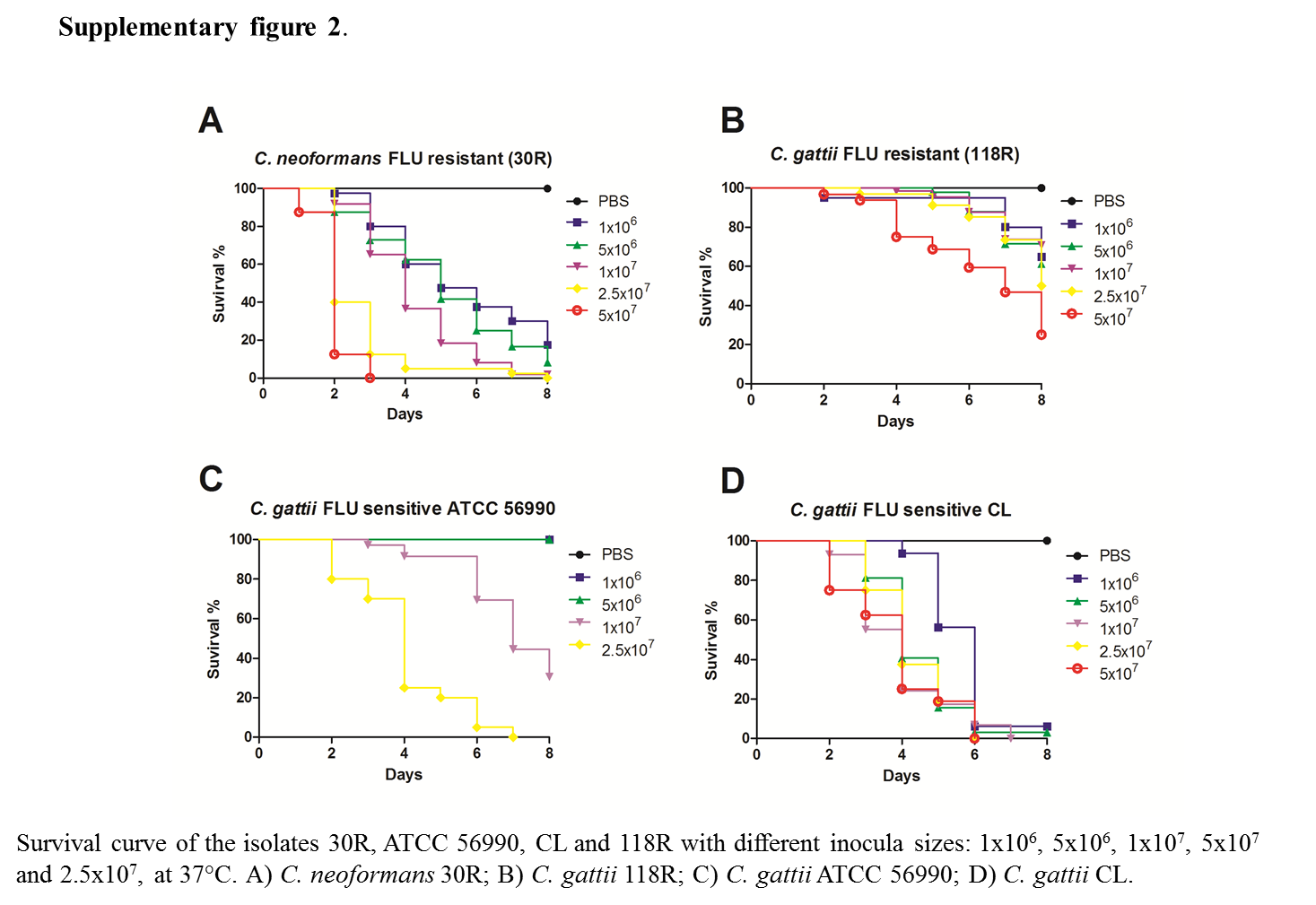

Supplement: Supplementary file 2 [file Image_2.TIF]
